# Rheological non-Newtonian behaviour of ethylene glycol-based Fe_2_O_3 _nanofluids

**DOI:** 10.1186/1556-276X-6-560

**Published:** 2011-10-25

**Authors:** María Jose Pastoriza-Gallego, Luis Lugo, José Luis Legido, Manuel M Piñeiro

**Affiliations:** 1 Departamento de Física Aplicada, Facultade de Ciencias, Universidade de Vigo, E-36310 Vigo, Spain

**Keywords:** nanofluid, rheology, hematite, ferrofluids, storage modulus, loss modulus, nanoparticles, shear thinning

## Abstract

The rheological behaviour of ethylene glycol-based nanofluids containing hexagonal scalenohedral-shaped *α*-Fe_2_O_3 _(hematite) nanoparticles at 303.15 K and particle weight concentrations up to 25% has been carried out using a cone-plate Physica MCR rheometer. The tests performed show that the studied nanofluids present non-Newtonian shear-thinning behaviour. In addition, the viscosity at a given shear rate is time dependent, i.e. the fluid is thixotropic. Finally, using strain sweep and frequency sweep tests, the storage modulus *G*', loss modulus *G*″ and damping factor were determined as a function of the frequency showing viscoelastic behaviour for all samples.

## Introduction

Research on nanofluids characterization has progressed remarkably in the last decade [1-8]. The first studies were performed at the US Argonne National Laboratories reporting anomalous thermal conductivity enhancements, beyond the prediction of classic models. Nowadays, it is well known that this effect depends on particle size, concentration, nature of base fluids, pH, temperature and nanoparticles clustering [5,7]. Moreover, it has been shown that other transport properties exhibit also unusual behaviour, including viscosity and rheological properties [9-12]. This means that the thermophysical profile of a nanofluid may be tuned to meet the requirements for a given industrial application. From a practical point of view, this offers numerous benefits [8,13,14] as improved heat transfer and stability, microchannel cooling without clogging, or reduction in required pumping power. Thus, nanofluids have emerged as suitable tailored working fluids in industrial, engineering and medical applications [5,13-15], but this requires a rigorous analysis of heat transfer and rheological properties. The effective viscosity of a nanofluid constitutes a key property as it governs the ease of flow, pressure drop and thus the pumping power involved during flow applications [16]. Concerning rheological behaviour of nanofluids, only a reduced number of studies can be found (see, e.g. Prasher et al. [17], Kwak and Kim [18], Chen et al. [2,9,19], Rao [20], Namburu et al. [21], Chevalier et al. [11]), evidencing a gap where further studies concerning rigorous characterization of their Newtonian behaviour limits and their viscoelastic trend are necessary. Moreover, recent studies have identified nanoparticle structuring/aggregation as a dominant mechanism for the thermal conductivity enhancement of nanofluids, and rheological analysis can provide a useful insight on their structure [2].

Following our previous research on nanofluids [22-25], we present experimental evidence of non-Newtonian behaviour of nanofluids obtained by dispersing hematite (Fe_2_O_3_) nanoparticles in ethylene glycol (EG). These ferrofluids are termed as smart functional fluids, due to some of its unique features, manifesting simultaneously fluid and magnetic properties, and have found applications in mechanical engineering, aerospace and bioengineering [26,27]. The selected base fluid, EG, constitutes an excellent benchmark to compare viscosity results with literature. As an example of rheological analysis of ferrofluids, Hong et al. [26] studied water-based Fe_3_O_4 _nanofluids, reporting shear-thinning behaviour.

## Experimental

In this work, homogeneous and stable suspensions of commercial hexagonal scalenohedral-shaped α-Fe_2_O_3 _(hematite) nanoparticles in EG were prepared at concentrations up to 25% in mass fraction (6.6% in volume fraction). The average nanoparticle diameter value determined was 29 ± 18 nm. More details about nanofluid preparation, stability and characterization have been recently reported [25]. These nanofluids were subjected to rheological analyses using a Physica MCR 101 rheometer (Anton Paar, Graz, Austria). The equipment allows to control torques between 0.5 μN·m and 125 mN·m and normal force from 0.1 to 30 N. The cone-plate geometry with a cone diameter of 25 mm and a cone angle of 1° was used. All experiments are conducted at a constant gap of 0.048 mm, and an initial stabilization period of 100 s is given for achieving constant temperature (303.15 K) using a Peltier system. Three replicates at each experimental condition were carried out.

## Results and discussion

With the aim to check the operation of this rheometer using a cone-plate geometry and at shear rates up to 1,000 s^-1 ^in the flow curves, initial experiments based on flow curves at controlled shear stress were carried out for pure EG, diisodecyl phthalate (DiDP) and polyalpha olefin (PAO-40). DiDP and PAO-40 represent Newtonian reference materials [28] in the moderate- to high-viscosity region. If compared with literature [10,25,29-32], excellent agreement is obtained for viscosities, with average deviations of 1.5%, 1.1% and 0.8% for EG [10,25,32], DiDP [30,31] and PAO-40 [29], respectively.

The rheological studies were performed under two types of flow [33,34]. The first is a non-linear viscoelastic experiment, the flow curve, or measurement of shear viscosity (*η*) as a function of shear rate ($\overset{{}^{\circ}}{\gamma }$). The second is the linear viscoelastic oscillatory experiment, leading to the determination of frequency-dependent energy storage modulus *G*' (elastic) and loss modulus *G*″ (viscous), which reveal the mechanical properties of the material under small amplitude oscillatory shear. Oscillatory shear measurements within the linear viscoelastic domain, intended to measure *G*' and *G*″, represent a useful way of characterizing complex fluids.

**Figure 1:**
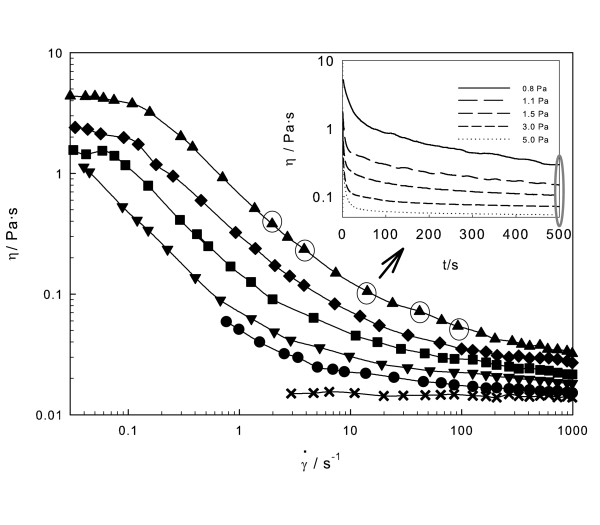
**Viscosity vs. shear rate ($\overset{{}^{\circ}}{\gamma }$) dependence of EG/Fe**_**2**_**O**_**3 **_**nanofluids**. At 303.15 K and *t *= 500 s for different weight concentrations: multiplication sign, EG; solid circle, 5 wt.%; solid inverted triangle, 10 wt.%; solid square, 15 wt.%, solid diamond, 20 wt.% and solid triangle, 25 wt.%. The inset shows viscosity vs. time dependence for 25 wt.% nanofluids at controlled shear stress.

Figure 1 shows the viscosity of EG vs. $\overset{{}^{\circ}}{\gamma }$ at 303.15 K, obtained from controlled shear stress tests. The applied torques start from 0.1 μN·m, covering a wide range of $\overset{{}^{\circ}}{\gamma }$ (3 to 1,000 s^-1^). Shear viscosity is independent of $\overset{{}^{\circ}}{\gamma }$, indicating Newtonian behaviour for EG. The flow curves of the EG/Fe_2_O_3 _nanofluids are also plotted, showing shear-thinning (pseudoplastic) non-Newtonian behaviour. As concentration rises, a Newtonian plateau with shear thinning appears in the lowest $\overset{{}^{\circ}}{\gamma }$ region, except for the lower concentration, due to the minimum $\overset{{}^{\circ}}{\gamma }$ threshold value of the rheometer used. The shear-thinning behaviour has also been observed by Hong et al. [26] for water/Fe_3_O_4 _ferrofluids, He et al. [35] and Tseng and Lin [36] for water/TiO_2 _nanofluids or Chen et al. [9] for EG/titanate nanotube nanofluids. However, this behaviour is completely different to the observed Newtonian one of EG/TiO_2 _[10] or propylene glycol/Al_2_O_3 _[17] nanofluids. While in some papers Newtonian behaviour has been supposed *a priori*, these results evidence that rheological tests must be always carried out to support such an affirmation. Most commercial viscometers work at fixed $\overset{{}^{\circ}}{\gamma }$ (≈100 s^-1^), and in these cases, the existence of unnoticed shear thinning may lead to a trend in the experimental viscosity measurements that might be erroneously attributed to the appearance of an anomalous enhancement.

Shear thinning of well-dispersed suspensions can be linked to the modifications in the structure and arrangement of interacting particles [37]. Shearing may cause the particles to orient in the direction of flow and its gradient. This can break agglomerates and hence reduce the amount of solvent immobilized by the particles. The interaction forces may then decrease and cause a lowering in the flow resistance and the apparent viscosity of the system.

The inset in Figure 1 shows the time evolution of shear viscosity for the 25 wt.% EG/Fe_2_O_3 _sample, and its decrease evidences thixotropic behaviour or a structure loss under shear. For this reason, all flow curves were measured after the preliminary application of a constant stress during 500 s. This time evolution of viscosity had not been reported for nanofluids so far, but it must be considered when performing viscosity measurements of nanofluids because it may also produce spurious trends for the measured data.

**Figure 2:**
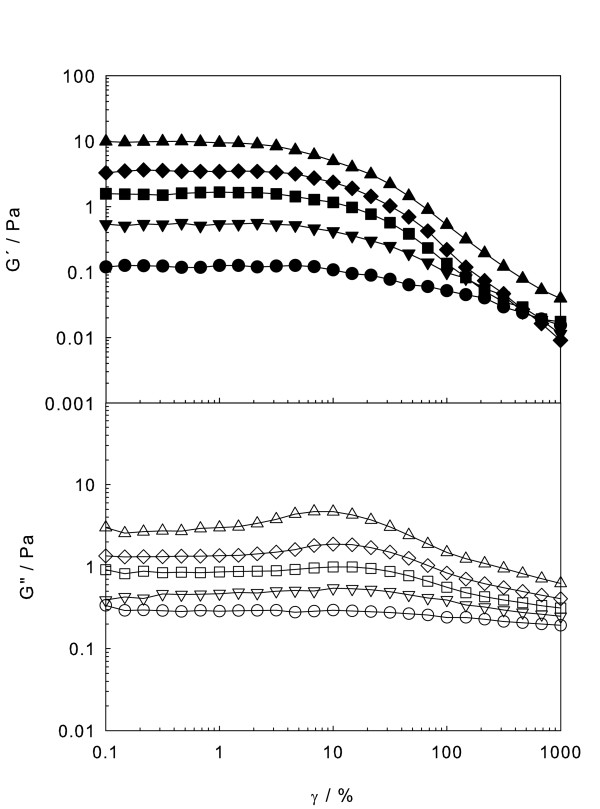
**Storage (*G*') and loss (*G*″) moduli**. As a function of strain at 10 rad/s and 303.15 K for different concentrations: (solid circle, empty circle) 5 wt.%, (solid inverted triangle, empty inverted triangle) 10 wt.%, (solid square, empty square) 15 wt.%, (solid diamond, empty diamond) 20 wt.% and (solid triangle, empty triangle) 25 wt.%.

**Figure 3:**
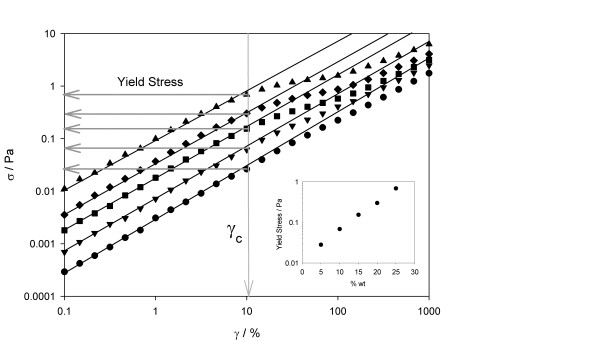
**Shear stress (*σ*) as function of strain (*γ*) at 10 rad s^-1 ^for different concentrations**. Solid circle, 5 wt.%; solid inverted triangle, 10 wt.%; solid square, 15 wt.%; solid diamond, 20 wt.% and solid triangle, 25 wt.%. The inset shows yield stress as a function of the nanoparticles concentrations.

The following step was performing oscillatory or dynamic experiments to determine the viscoelastic behaviour. The power of dynamic testing is that stress can be separated into its elastic and viscous contributions, and the elastic or storage modulus *G*' and the viscous or loss modulus *G*″ can be calculated. First, strain sweep tests at constant *ω *= 10 rad s^-1 ^were carried out (cf. Figure 2) to identify the linear viscoelastic region in the strain range from 0.1% to 1,000%. The linear regime, where *G*' and *G*″ are constant regardless of strain amplitude, is clearly observed. *G*' decreases monotonically as strain increases (Figure 2, upper panel), while *G*″ goes through a maximum, exhibiting an overshoot phenomenon (Figure 2, lower panel). The interpretation is that when an external strain is imposed, the structure of nanofluids resists the deformation up to a certain strain, where *G*″ increases, and then the structure is lost by the disaggregation of nanoparticles due to large deformations over the critical strain, after which the nanoparticles align with the flow field, decreasing *G*' and *G*″. This effect, more important at higher concentrations [25,38,39], may be governed by the aggregates dimension and radius of gyration. In order to evidence the well-defined linear viscoelastic range of this test, carried out at 10 rad s^-1^, the stress-strain curves are also displayed in Figure 3, for all concentrations. The critical strain is shown to be independent of concentration, while the stress upper limit of the linear viscoelastic regime region increases linearly with concentration, as shown in the inset of the figure.

**Figure 4:**
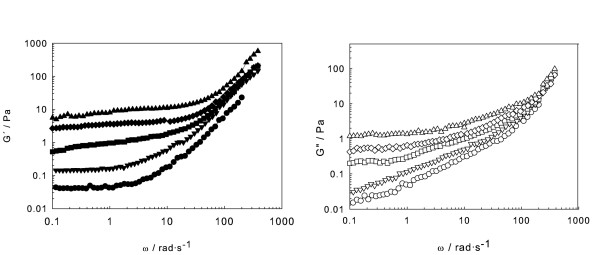
**Storage (*G*') and loss (*G*″) moduli**. As a function of frequency for EG/Fe_2_O_3 _nanofluids at 1% strain: (solid circle, empty circle) 5 wt.%; (solid inverted triangle, empty inverted triangle) 10 wt.%; (solid square, empty square) 15 wt.%, (solid diamond, empty diamond) 20 wt.% and (solid triangle, empty triangle) 25 wt.%.

Then, frequency sweep tests were carried out in the linear viscoelastic region, with angular frequencies ranging from 0.1 to 400 rad s^-1^, with a constant strain value of 1%. The experimental data of storage (elastic) and loss (viscous) moduli are shown in Figure 4. The storage modulus exceeds the loss modulus, *G*' >*G*″, especially for higher concentrations, and *G*' values are practically constant in the low frequency range, indicating a typical gel structure and the dominant elastic nature of the material under these conditions. However, for 5 wt.% concentration, a crossover frequency (*G*' = *G*″) appears, meaning that below 10 rad s^-1^, it shows liquid-like behaviour but changes beyond that value. Both moduli increase with concentration at a given constant frequency, they increase with frequency beyond an approximate value of 10 rad s^-1^, and almost all *G*″ values fall on a straight line for the highest frequencies. These results must be underlined because, starting from a base fluid that exhibits Newtonian behaviour, the addition of hematite nanoparticles produces, even at low concentrations and frequencies, a continuous transition towards elastic behaviour, which means that rheological studies of its viscoelastic nature become essential to determine its potential practical use for any technical application. Moreover, due to their magnetic nature, the influence of an external magnetic field in the viscoelastic behaviour of this nanofluid becomes an attractive topic.

**Figure 5:**
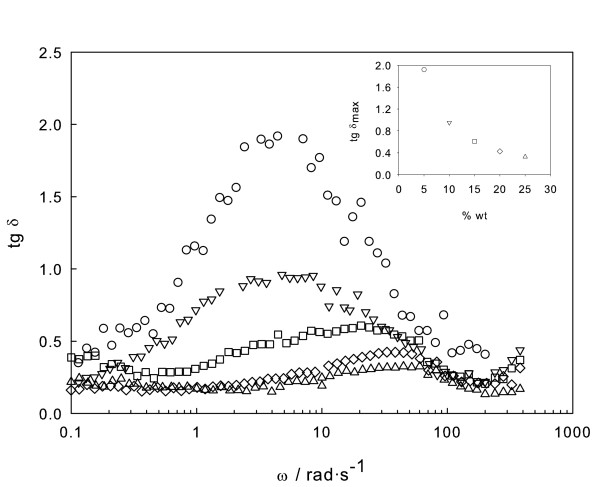
**Damping factor (tg *δ*)**. As a function of frequency at 1% strain for various concentrations of EG/Fe_2_O_3 _nanofluids: empty circle, 5 wt.%; empty inverted triangle, 10 wt.%; empty square, 15 wt.%; empty diamond, 20 wt.%; and empty triangle, 25 wt.%. The inset shows maximum damping factor as a function of the weight concentration of nanofluids.

Finally, the trend of *G*″/*G*' (tangent of the phase angle *δ*, denoted as damping factor) with frequency was determined (Figure 5). For metals, the damping factor is typically small (approximately 0.0005), whereas for viscoelastic materials, it may exceed the unity. This damping factor is shown to decrease as concentration rises, corresponding to a *G*' increase, or an evolution from fluid towards elastic behaviour, as stated. Another remarkable feature is the presence of a well-defined maximum in the damping factor, appearing at frequencies shifting to higher values with sample concentration, and its height decreases exponentially with concentration.

## Conclusions

This work evidences the non-Newtonian nature of EG/Fe_2_O_3 _nanofluids, showing shear thinning and thixotropy. All samples show viscoelastic nature, suggesting that a combination of particle aggregation and shape effects is the mechanism for its high-shear rheological behaviour, which is also supported by the thermal conductivity measurements [7,24,39]. *G*' decreases after a certain critical strain, and *G*″ presents an overshoot phenomenon. Finally, the results of the frequency sweep show that the damping factor presents a maximum against frequency, corresponding to a continuous evolution with concentration from viscous to elastic nature. This is an evidence of important aggregation and structural changes in the samples, a subject still poorly studied that deserves further attention.

## Competing interests

The authors declare that they have no competing interests.

## Authors' contributions

MJPG performed the nanofluid samples characterization and experimental measurements. LL contributed with the selection of the optimal experimental setting of the rheometer and type of tests to be performed, and coordinated the redaction of the manuscript. JLL participated in the critical evaluation of experimental results. MMP conceived the study, and participated in its design and coordination. All authors read and approved the final manuscript.
